# [Expression of Concern] Somatic mutations in myeloid cell leukemia-1 contribute to the pathogenesis of glioma by prolonging its half-life

**DOI:** 10.3892/mmr.2026.14066

**Published:** 2026-11-09

**Authors:** Xiupeng Lv, Guang Tan, Yiqun Yao, Li Lv, Xiaoqin Deng, Lei Dong, Shuang Li, Linlin Li, Yinghui Xu

Mol Med Rep 12: 1265-1271, 2015; DOI: 10.3892/mmr.2015.3493

Subsequently to the publication of the above article, a concerned reader has drawn the Editor's attention to the fact that they have identified a large number of major inconsistencies involving the reported sequencing strategy, mutation identities, experimental conditions, figure labeling and statistical analysis in this paper, which collectively would serve to undermine both the authenticity and the reproducibility of the results contained therein. Among the most significant concerns was the inconsistency between the genes reportedly screened and the mutations reported: In the Materials and methods section, it was stated that the authors screened the coding regions of 20 specified cancer-associated genes, although Table II reported mutations in ZMYM3, SORCS1, and ASAP3, none of which were included among those 20 genes. In addition, the paper reported an apparently impossible PCR and sequencing strategy, together with implausible experimental conditions for a number of the other experimental methods, the use of an inappropriate reference database for nuclear genes, inconsistent mutation identities and numbering, contradictory interpretations of the data in Figs. 2 and 3, and incorrect labeling and insufficient validation of the data in Fig. 4.

The authors have been contacted by the Editorial Office to offer an explanation for these potential anomalies and inconsistencies in the reporting of this study, and we are awaiting their response. Owing to the fact that the Editorial Office has been made aware of potential issues surrounding the scientific integrity of this paper, we are issuing an Expression of Concern to notify readers of these problems while the Editorial Office continues to investigate this matter further.

